# Number of years of participation in some, but not all, types of physical activity during adolescence predicts level of physical activity in adulthood: Results from a 13-year study

**DOI:** 10.1186/s12966-015-0237-x

**Published:** 2015-06-10

**Authors:** Mathieu Bélanger, Catherine M. Sabiston, Tracie A. Barnett, Erin O’Loughlin, Stéphanie Ward, Gisèle Contreras, Jennifer O’Loughlin

**Affiliations:** Department of Family Medicine, Université de Sherbrooke, Sherbrooke, Canada; Centre de formation médicale du Nouveau-Brunswick, 18 rue Antonine Maillet, Pavillon J.-Raymond-Frenette, Moncton, NB E1A 3E9 Canada; Office of research services, Vitalité Health Network, Moncton, Canada; Faculty of Kinesiology and Physical Education, University of Toronto, Toronto, Canada; Epidemiology and Biostatistics Unit, INRS-Institut Armand-Frappier, Université du Québec, Laval, Canada; Centre de recherche du CHU Sainte-Justine, Montréal, Canada; Centre de recherche du Centre hospitalier de l’Université de Montréal (CRCHUM), Montréal, Canada; Department of INDI & Exercise Science, Concordia University, Montreal, Canada; Faculty of medicine and health sciences, Université de Sherbrooke, Sherbrooke, Canada; Département of Social and Preventive Medicine, Université de Montréal, Montréal, Canada; Institut national de santé publique du Québec (INSPQ), Montréal, Canada

**Keywords:** Physical activity groupings, Sustainability of physical activity, Cohort study, Adolescence

## Abstract

**Background:**

Adolescent physical activity (PA) levels track into adulthood. However it is not known if type of PA participated in during adolescence is associated with PA levels later in life. We aimed to identify natural groupings of types of PA and to assess whether number of years participating in these different groupings during adolescence is related to PA level in early adulthood.

**Methods:**

673 adolescents in Montreal, Canada, age 12–13 years at baseline (54 % female), reported participation in 29 physical activities every 3 months over 5 years (1999–2005). They also reported their PA level at age 24 years (2011–12). PA groupings among the 29 physical activities were identified using factor analysis. The association between number of years participating in each grouping during adolescence and PA level at age 24 was estimated using linear regression within a general estimating equation framework.

**Results:**

Three PA groupings were identified: “sports”, “fitness and dance”, and “running”. There was a positive linear relationship between number of years participating in sports and running in adolescence and PA level at age 24 years (β (95 % confidence interval) = 0.09 (0.04-0.15); 0.08 (0.01-0.15), respectively). There was no relationship between fitness and dance in adolescence and PA level at age 24.

**Conclusions:**

The association between PA participation in adolescence and PA levels in young adulthood may be specific to certain PA types and to consistency of participation during adolescence. Results suggest that efforts to establish the habit of participation in sports and running in adolescence may promote higher PA levels in adulthood.

## Background

In Canada, 85 % of adults and over 90 % of youth do not attain the recommended minimum of 150 min of moderate to vigorous physical activity (PA) per week [1, 2]. Physical inactivity is also highly prevalent in other developed countries [3, 4], and therefore represents one of today’s leading public health challenges [5]. It is estimated that physical inactivity is responsible for 6 % of coronary heart disease, 7 % of type 2 diabetes, 10 % of breast cancer, 10 % of colon cancer, and 9 % of premature mortality worldwide [6]. Since PA tracks over time [7], interventions that increase PA at a young age may promote healthy levels of PA participation life-long. It is well-accepted that early intervention is needed to slow the marked declines in PA that are typical during adolescence [8, 9]. To guide the conceptualization of such intervention, it may be helpful to identify which types of physical activities are more likely to be associated with adult PA and to allocate limited public health resources to support such activities. However, how specific types of PA in youth relate to PA levels in adulthood is not well understood.

Based on the extant literature, there is some evidence that early participation in a specific type of PA increases the likelihood of participating in that same PA in adulthood [10]. In one study, participation in a specific PA (i.e., soccer, waterskiing, alpine skiing, cross-country skiing, judo, power sports, swimming, ball games, cycling, jogging, aerobics, bodybuilding), at age 15 years more than doubled the odds of participation in that same PA at age 23 years [10]. Also, early participation in specific types of PA is associated with higher levels of PA in adulthood. For example, although it does not necessarily prevent the decline in PA during adolescence [11], youth participation in organized PA such as hockey, soccer, volleyball, martial arts, track and field, dancing and gymnastics is associated with higher PA levels in adulthood [12–15]. Similarly, a Finnish study showed that in men, participation in endurance sports and martial arts in adolescence was associated with higher PA levels at age 31 years. In women, running, gymnastics and horseback riding in adolescence were positively associated with adult PA [13]. Another study found weak but significant positive associations between number of school sports, organised sports teams, and number of physical education classes in adolescence and total amount of discretionary PA in adulthood [16]. However, these studies are limited by a single assessment of PA type in youth that is measured over relatively brief [16] or unspecified [13–15] time periods.

In addition to the link between participation in similar types of PA in adolescence and adulthood [12–15, 17], different types of PA have different likelihoods of being maintained throughout adolescence [18, 19]. For example, 90 % of youth who reported participation in individual-based PA in early adolescence sustained participation in this type of PA over five years, whereas the percentage that sustained team-based PA was 55 % [18]. Researchers have also shown that consistently reporting being among the most physically active, across five survey cycles separated by three year intervals, in childhood and adolescence is associated with a higher likelihood of being among the most active in adulthood [20]. However, it is not yet clear if consistent participation in a given PA during adolescence is associated with PA levels in adulthood.

In order to improve lifelong PA levels, we aimed to improve understanding of whether long-term participation in different PA types in adolescence is associated with PA level in adulthood. Identifying PA groupings however is challenging since PA can be characterized according to numerous different features (i.e., intensity, purpose, setting, organisation, competitiveness, etc.). It may therefore be more informative to group activities that are generally similar according to a combination of these characteristics. The objectives of this current study were i) to identify natural groupings of types of PA in adolescents, and ii) to investigate whether number of years participating in these different groupings during adolescence is related to PA levels in young adulthood.

## Methods

### Study population

Data for this analysis were available in the Natural History of Nicotine Dependence in Teens (NDIT) study, a prospective investigation of 1293 students initially aged 12–13 years. Data collection began in fall 1999 and was repeated every 3 months during the 10-month school year for the first 5 years of the study, for a total of 20 survey cycles. Cycle 21 took place in 2007–08 and cycle 22 took place in 2011–12 (a median of 6.2 years after the last cycle in high school) when participants were age 24 years on average. In cycles 1–20, participants completed self-report questionnaires in French or English, in a classroom setting or as a larger group in the school cafeteria. Data collectors visited schools twice during each cycle, so that participants who were absent on the first day could complete the questionnaire on the second day. In cycle 22, participants completed a self-report questionnaire administered in the NDIT research offices. A detailed description of NDIT methods is available elsewhere [21]. The current analysis was restricted to participants who provided data in at least four of the first five years of data collection, and in cycle 22. All participants provided assent at the time of recruitment, and a parent or guardian provided signed informed consent. Consent was provided by participants in cycles 21 and 22, since they had attained legal age (18 years). The study received ethics approval from the Institutional Review Boards at McGill University and the Centre de recherche du Centre hospitalier de l’Université de Montréal.

### Study variables

In cycles 1–20, number of PA sessions per week was obtained in a 7-day recall adapted from the Weekly Activity Checklist [22] to reflect common activities engaged in by adolescents in Montreal. At each cycle, adolescents were asked: “Think about the physical activities that you did last week from Monday to Sunday outside your regular gym class at school. For each activity that you did for 5 min or more at one time, mark an “X” to show the day(s) on which you did that activity…,” followed by a list of 29 activities. The 5-min criterion is supported by findings that moderate to vigorous intensity PA sessions of this duration protect against obesity in youth [23]. In cycle 1, the PA recall checklist was administered twice in a period of two weeks in a subsample of 76 students in one school. The correlation between the two administrations in reported level of involvement in PA was *r =* 0.73, which is comparable to the test retest reliability of accelerometers in similar populations [24].

In cycle 22, participants reported their PA level using the International Physical Activity Questionnaire short form (IPAQ-SF). The IPAQ-SF includes 6 items that estimate time spent in vigorous intensity activities, moderate intensity activities (not including walking), and walking over the last 7 days. Specifically, vigorous PA was measured in two items: “During the last 7 days, on how many days did you do vigorous physical activities (e.g., heavy lifting, digging, aerobics, fast bicycling) for at least 10 min at a time?” and “On the days that you did vigorous physical activities, how many minutes did you usually do per day?” Time spent in moderate PA and walking were obtained using similar questions and each was multiplied by the estimated intensity in METs. METs across activities were summed to obtain a weekly estimate of PA (MET minutes per week) as described in the IPAQ data processing guide (www.ipaq.ki.se). One MET represents the energy expended while sitting quietly at rest and is equivalent to an oxygen consumption of 3.5 ml/kg/min [25].

### Analyses

The maximum level of participation reported for each specific PA in the PA checklist over the first four cycles (i.e., during grade 7) was used to represent level of involvement in a given activity in the first year of data collection. We excluded indoor and outdoor chores, which were deemed non-leisure and nondiscretionary. We also excluded walking since it was reported by nearly all participants at each cycle. Exploratory factor analysis (EFA) was used to identify natural groupings among the 26 remaining PAs in the checklist using the FACTOR procedure in SAS version 9.2 (SAS Institute Inc., Cary, NC, USA.). An oblique rotation after an orthogonal varimax rotation was used [26]. EFA was performed on year-1 data using half the sample, and then EFA was repeated in the second half of the sample. Both analyses yielded similar distributions of PA over the same number of factors, and therefore identification of PA groupings was conducted using the complete sample. We decided on the number of factors to retain based on an examination of Scree plots and a minimum Eigenvalue of 1. PA groupings did not include variables that loaded weakly (i.e., factor loading < 0.4) [27]. After PA groupings were identified, participants were categorized as having reported involvement in each PA grouping over 0, 1, 2, 3, 4, or 5 years during high school.

Because the IPAQ scores obtained in cycle 22 (i.e., the outcome of interest) were skewed, a logarithmic transformation of the data was undertaken which resulted in the scores approximating a normal distribution. Multivariable linear regressions were used to estimate the effect of number of years participating in different PA groupings during adolescence on PA level in early adulthood. Separate models were fitted for each PA grouping, and one model included all three PA groupings. The models were adjusted for age, sex, and parents’ education (one or both parents attended university, neither parent attended university). For the main analyses, number of years participated in a PA grouping was treated as an interval variable. However, we also modeled the exposure as a series of dummy variables for the creation of figures. We used the generalized estimating equations framework to account for clustering at the school level (GENMOD procedure in SAS).

## Results

**Fig. 1:**
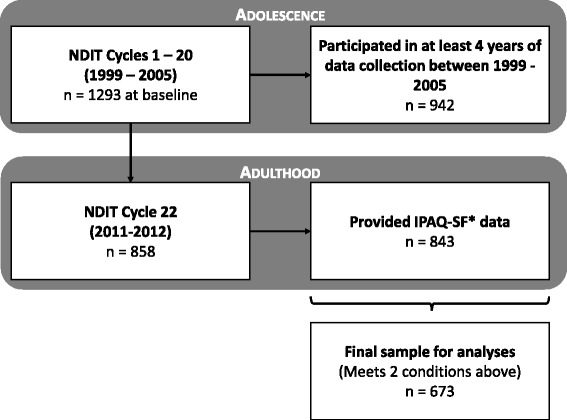
Description of the natural history of the Nicotine Dependence in Teens (NDIT) Study and sample retained for current analyses

A total of 858 participants provided IPAQ data at age 24 years. Of these, 15 had improbably high values and were excluded as recommended in the IPAQ data processing guide (www.ipaq.ki.se). Among the 843 with valid IPAQ data, 673 had data in at least four of the five years of follow-up during high school and were retained for analysis (Fig. 1). Half (54 %) were girls, 62 % had a university-educated parent, and the mean (standard deviation) age of participants at cohort inception was 12.7 (0.4) years (Table 1). The median PA level of participants at cycle 22 was 1716 (interquartile range: 834 to 3210) MET-minutes per week. No meaningful differences in sex, parental education, age or median PA level at cycle 22 were observed between participants retained and those excluded from the analyses (all *p* > 0.3).

**Table 1:** Characteristics of the 673 study participants retained for analyses

Sex	
Boys, n (%)	310 (46)
Girls, n (%)	363 (54)
Age at baseline, years, mean ± SD years	12.7 ± 0.4
Parental education	
Neither parents has university education, n (%)	258 (38)
At least one parent has university education, n (%)	415 (62)
PA level at cycle 22, median (interquartile range) MET-minutes per week	1716 (834 to 3210)

Three PA groupings were identified in the EFA (Table 2). The first PA grouping was labelled “sports” and included eight sport-oriented activities including ice hockey, ice skating, football, roller blading/skateboarding, basketball, soccer, boxing and baseball (i.e., activities governed by a set of rules that can be engaged in competitively [28]). The second grouping was labelled “fitness and dance” and included four activities including jazz dance, dancing, gymnastics and games. The final grouping was labelled “running” and included running and mixed walking, which represents activities such as hiking and walking mixed with jogging. Long-term involvement in a given grouping was not strongly related to long-term involvement in another grouping (Spearman rank correlation coefficients between number of years participating in the sports and fitness groupings was r = 0.1; r = 0.3 between sports and running; and r = 0.3 between the fitness and running). All PA groupings could therefore be included in the same multivariate model.

**Table 2:** Summary of exploratory factor analysis results for groupings of physical activity using maximum likelihood estimation and varimax rotation

	Participants reporting the activity in Year 1 (%)^b^		Factor loadings	
		Factor 1	Factor 2	Factor 3
		Sports	Fitness and dance	Running
Ice hockey	35	0.648	−0.135	0.070
Ice skating	31	0.534	0.073	0.098
Football	29	0.532	0.113	0.050
Roller blading/ skateboarding	51	0.502	0.100	0.169
Basketball	55	0.490	0.070	0.088
Soccer	50	0.425	0.063	0.239
Boxing	28	0.407	0.085	0.153
Baseball	18	0.402	0.071	0.046
ªBicycle	63	0.392	0.133	0.182
ªRacket sports	30	0.375	0.103	0.048
ªDownhill ski/ snowboard	25	0.327	0.018	0.135
ªVolleyball	18	0.317	0.193	0.009
ªBall playing	38	0.307	0.216	0.184
ªKarate	13	0.285	0.103	0.108
ªSwimming	40	0.278	0.219	0.003
ªCross-country ski	10	0.226	0.049	0.058
Jazz dance	12	−0.023	0.491	0.094
Dancing	40	−0.054	0.567	0.065
Gymnastics	18	0.145	0.447	0.018
Games	36	0.129	0.417	0.254
ªJump rope	19	0.162	0.358	0.129
ªExercise	69	0.350	0.351	0.330
ªTrack and field	21	0.164	0.310	0.203
Running	59	0.236	0.169	0.685
Mixed walking	70	0.083	0.164	0.623
ªOutdoor play	47	0.254	0.198	0.333
Eigenvalues		6.13	1.92	1.04
% of variance		23.6	7.4	4.0

ªNot included in the main analysis because of low (<0.4) factor loading

^b^The proportion of boys and girls reporting participation in each activity in each of the first 5 years of study is reported in Belanger M, Gray-Donald K, O’Loughlin J, Paradis G, Hanley J. When adolescents drop the ball: sustainability of physical activity in youth. Am J Prev Med 2009, 37:41–49

There were small but statistically significant differences in the distributions of boys and girls participants by number of years participating in running (Table 3). However, a higher proportion of boys than girls reported long-term participation in sports, while a higher proportion of girls reported long-term participation in fitness and dance. The proportion of participants with at least one university-educated parent was higher among participants who sustained sports and running, but lower among participants who sustained fitness and dance.

**Table 3:** Percent of participants according to number of years of participation in sports, fitness and dance and running during the five years of high school, *n* = 673

	Number of years						
	0	1	2	3	4	5	
	%	%	%	%	%	%	*p* ^a^
Sports							
Total	10	10	13	12	21	35	
Boys	5	4	5	8	25	54	
Girls	14	15	19	15	17	20	<.0001
Fitness and Dance							
Total	36	23	14	12	7	9	
Boys	46	26	11	9	3	5	
Girls	26	20	17	14	11	12	<.0001
Running							
Total	8	11	17	16	18	30	
Boys	7	8	15	18	20	31	
Girls	9	14	18	13	16	30	0.046

^a^Chi square test (5 *df*) on difference of distribution between boys and girls

**Fig. 2:**
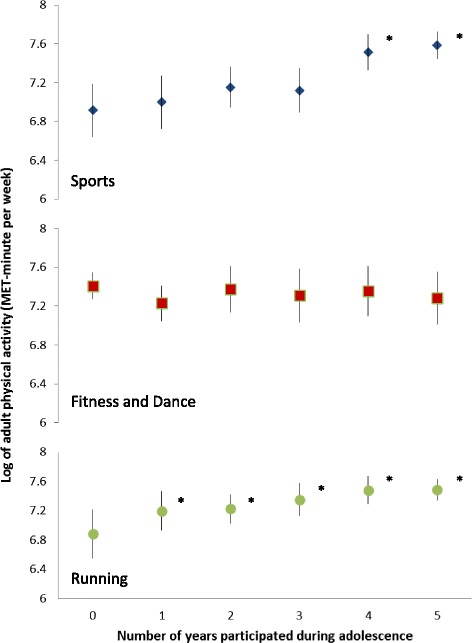
Log of adult physical activity level in relation to number of years of participation in three physical activity groupings during adolescence

Number of years participating in two PA groupings - sports and running - in adolescence were statistically significantly and positively associated with adult PA levels (Table 4). All significant univariate associations remained statistically significant after adjustment for covariates. Participants with 4–5 years of sports involvement in adolescence reported statistically significantly higher levels of PA in adulthood than adolescents with no sports (Fig. 2). Adolescents who reported sports in one, two, or three years had adult PA levels similar to adolescents with no sport participation. In contrast, in comparison with no years running, more years running during adolescence was associated with higher levels of adult PA. There was no significant association between number of years participating in fitness and dance during adolescence and adult PA. Results were similar for analyses wherein a separate multivariate model was fitted for each PA grouping (results not shown).

**Table 4:** Beta coefficients (β) and 95 % confidence intervals (CI) estimated in multiple linear regressions for the log of adult physical activity level^a^ in relation to number of years participating in sports, fitness and dance and running during adolescence

	Univariate models^b^	Multivariate model^b^
	β (95 % CI)	β (95 % CI)
Female	−0.44 (−0.58, −0.41)**	−0.28 (−0.40, −0.15)**
Age (years)	−0.02 (−0.09, 0.14)	−0.02 (−0.16, 0.13)
Parent university-educated	−0.001 (−0.13, 0.14)	−0.04 (−0.18, 0.09)
No. years in sports	0.14 (0.09, 0.20)**	0.09 (0.04, 0.15)**
No. years in fitness and dance	−0.02 (−0.07, 0.02)	−0.02 (−0.06, 0.02)
No. years running	0.10 (0.02, 0.18)*	0.08 (0.01, 0.15)*

*p < 0.05; **p < 0.001

^a^Log of MET-minutes per week estimated from the International Physical Activity Questionnaire short form

^b^accounting for school-level clustering usingthe general estimating equation framework

## Discussion

In this study, physical activities commonly engaged in by adolescents were categorized into one of three groupings: sports, fitness and dance, or running. Adult PA levels were predicted by number of years participating in sports and running during adolescence, but not by number of years participating in fitness and dance. Whereas more years of participation in running during adolescence was associated with higher adult PA levels, attaining a minimum threshold of involvement in sports during adolescence was needed to favour higher adult PA levels. These results support the importance of consistency in participation over the adolescent years. This is the first study to document a relationship between duration of involvement in natural PA groupings during adolescence and adult PA levels.

Although only 7 % of youth attain current PA guidelines [1], over half of Canadian youth take part in organized sports [29]. Our findings suggest that sport participation must be sustained for at least four years during high school in order to translate into higher adult PA levels. Specifically, the average PA level in adults was similar among those who had never participated in sports during adolescence and those who took part in sports for up to three years. This finding may relate to barriers to sport participation during adolescence persisting into adulthood, which would be consistent with studies reporting similar barriers in both age-groups [30–36]. It is also possible that interrupting participation in sports during adolescence indicates a shift to other (non-PA) activities which are sustained into adulthood and compete for discretionary time. Further, sport participation often requires use of sporting facilities as well as the formation of a team, which may not be as feasible in adulthood as it is in adolescence [37]. Similarly, those who do not sustain a specific PA during adolescence may have fewer opportunities to engage in this activity later because of access barriers.

Each additional year that adolescents participated in running was related to higher adult PA levels. Running is the basis for numerous types of PA. Our result is consistent with the notion that running can be maintained over time since it is easily transferable from one PA context to another [38]. Running also requires few motor skills, which makes the activity highly accessible regardless of ability. Moreover, running requires little in terms of structure, organisation or cost and equipment so that people can easily opt in or out. Number of years that running is sustained may therefore be a marker of intrinsic motivation for PA, which is a good predictor of future PA levels [39]. Further, running and walking represent active modes of transportation. It is therefore possible that adolescents who adopt active transportation early in life retain this habit as a way of life later on [40].

We found that adolescent fitness and dance related activities were not related to adult PA level. In a study among adults, participation in fitness-focused activities such as aerobics was less likely to be sustained over 10 weeks than participation in martial arts [41]. The authors attributed this difference to fitness activities often being performed because of extrinsic motivations such as wanting to improve body shape, muscle tone or weight (41, 43). Comprehensive systematic reviews also strongly suggest that extrinsic motivation for PA may be less conducive than intrinsic motivation to sustaining PA [39, 42]. Although we do not have information on levels or types of motivation for PA, it is possible that those who reported participation in fitness and dance during adolescence took part in PA because of external motivations, and were therefore less likely than internally motivated individuals to sustain participation. Another potential explanation is that participants in fitness and dance may face fewer opportunities for participation as they age given that such activities are more widely available for youth than for adults. These explanations remain speculative. More research is needed to understand the mechanisms underpinning variations in the relationships between different PA groupings during adolescence and adult PA. Further, although not associated with future PA, fitness and dance have previously been shown to be associated with other beneficial effects including increased aerobic capacity, improved body composition, greater bone density, muscular size and strength, improved psychological wellbeing, increased self-esteem and reduced anxiety [43–50].

An important strength of this study is that the frequent administration of questionnaires enabled us to create a measure of duration of participation in three natural PA groupings. However, this measure was based on self-report and did not account for the amount, intensity, regularity, or timing of participation. Additionally, the sport grouping does not distinguish between organised and unorganised sports, and it includes physical activity that may have been performed in non-sport contexts. Use of factorial analyses to identify PA groupings led to the exclusion of PAs that did not load onto one of the three factors, which introduced the potential for misclassification of number of years participants took part in PA during adolescence. However, sensitivity analysis including all 26 activities did not alter the findings. Finally, it should be recognised that consistency of participation in a PA grouping may include variation in the specific activity participated in within the same grouping.

## Conclusions

In conclusion, this study suggests that the association between adolescent and adult PA is dependent, at least in part, on the type of PA engaged in during adolescence. Further the relationship may relate to the total number of years and consistency of participation during adolescence. Specifically, adult PA levels were predicted by number of years participating in sports and running during adolescence, but not by participation in fitness and dance. Efforts to promote sustained participation in sports and running during adolescence may translate into higher PA levels in adulthood.
